# Prefrontal cortex iron content in neurodegeneration and healthy subjects: A systematic review

**DOI:** 10.1002/ibra.12195

**Published:** 2025-04-10

**Authors:** Sana Mohammadi, Sadegh Ghaderi, Masoud Hoseini Pourasl, Farzad Fatehi

**Affiliations:** ^1^ Neuromuscular Research Center, Department of Neurology, Shariati Hospital Tehran University of Medical Sciences Tehran Iran; ^2^ Department of Neuroscience and Addiction Studies, School of Advanced Technologies in Medicine Tehran University of Medical Sciences Tehran Iran; ^3^ Department of Radiology Kettering General Hospital Kettering UK

**Keywords:** cognitive aging, iron, magnetic resonance imaging, neurodegeneration, prefrontal cortex, quantitative susceptibility mapping

## Abstract

Iron accumulation in the prefrontal cortex (PFC) has been implicated in neurodegeneration and cognitive decline. Magnetic resonance imaging (MRI) enables noninvasive quantification of brain iron content and deposition. This review aimed to summarize the evidence on the MRI‐based assessment of PFC iron accumulation in healthy individuals and patients with neurodegeneration. A systematic preliminary literature review was conducted using the PubMed, Scopus, Web of Science, and Embase databases. MRI techniques for capturing susceptibility changes reflecting iron, such as susceptibility‐weighted imaging (SWI), quantitative susceptibility mapping (QSM), and R2* mapping, were included. Data were extracted, and narrative synthesis was performed. Twelve studies that measured PFC iron levels using MRI in diseases with neurodegeneration (five studies) and healthy subjects (seven studies) were included. In general, studies involving diseases with neurodegeneration have found that increased PFC iron content correlates with cognitive impairment. Aging studies on healthy subjects have reported that age‐related accumulation of PFC iron, particularly in the dorsolateral, medial, and anterior subregions, increases with age, and is associated with reduced dopamine signaling and poorer cognition. MRI techniques, such as QSM, can quantify prefrontal iron accumulation in diseases with neurodegeneration and aging. As imaging biomarkers, increased prefrontal iron levels may contribute to neurodegeneration and cognitive decline. Longitudinal studies combining advanced QSM and other advanced neuroimaging techniques with cognitive assessments may further elucidate the effects of iron dysregulation on PFC function. Thus, our findings highlight the importance of MRI as a sensitive tool for assessing PFC iron content and its potential role in understanding the pathogenesis of neurodegeneration and the effects of aging on the brain.

## Graphical abstract

This systematic review summarizes magnetic resonance imaging (MRI)‐based assessments of prefrontal cortex (PFC) iron accumulation in healthy individuals and patients with neurodegenerative diseases. The review includes 12 studies using techniques such as susceptibility‐weighted imaging (SWI), quantitative susceptibility mapping (QSM), and R2* mapping. Findings indicate that increased PFC iron content correlates with cognitive impairment in neurodegenerative diseases, with QSM identified as a potential biomarker for early cognitive changes and disease progression. In healthy aging, studies show age‐related iron accumulation in PFC subregions, associated with reduced dopamine signaling and poorer cognition. The review highlights MRI's role as a sensitive tool for assessing PFC iron content, emphasizing the need for longitudinal studies combining advanced MRI and cognitive assessments to further understand the effects of iron dysregulation on PFC function.

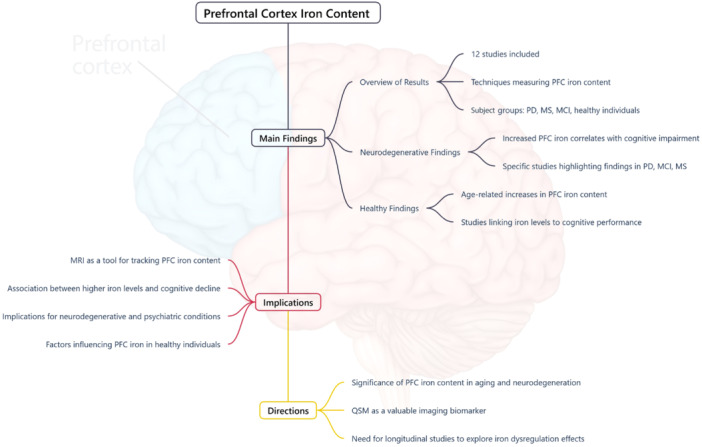

## 1 INTRODUCTION

In a preliminary 1958 study by Hallgren and Sourander, a landmark postmortem examination was conducted to investigate iron accumulation in the aging brain.^1^ This study revealed an increase in nonheme iron concentration with age across all measured brain regions, including the cerebral cortex. Notably, in the prefrontal cortex (PFC), there was little or no increase in iron levels after 30 years of age. However, iron is crucial for many biological functions, such as transporting oxygen, synthesizing DNA, and facilitating cellular respiration.^2^, ^3^ In the human brain, iron plays a crucial role in neuronal development,^4^, ^5^ myelination,^6^ and neurotransmitter synthesis,^7^, ^8^ among other functions.^9^ Aberrant iron has been associated with several diseases with neurodegeneration, such as Parkinson's disease (PD),^10^ multiple sclerosis (MS),^11^ amyotrophic lateral sclerosis (ALS),^12^, ^13^ and mild cognitive impairment (MCI)^14^, ^15^ and so on.

Iron, an essential element for various brain functions, accumulates in the brain at the age of years.^16^ This accumulation is particularly evident in regions such as the basal ganglia, substantia nigra, and frontal white matter.^17^ While nonheme iron concentration is relatively low in the neocortex, including the PFC, compared to the basal ganglia, its role in cortical T2* measurements, a marker for iron content, is significant.^17^, ^18^ The PFC is a brain region that is particularly vulnerable to iron deposition.^19^, ^20^ This region is critical for cognitive functions such as attention, working memory, and decision‐making.^21^, ^22^ Iron accumulation in the PFC may be associated with various neurological and neuropsychiatic disorders.^20^, ^23^ Thus, understanding the iron content in the brain can provide valuable information on the pathogenesis of these conditions and potentially guide diagnostic management.^24^, ^25^

Magnetic resonance imaging (MRI) has emerged as a powerful, noninvasive tool for quantification of brain iron content.^26^ Various conventional and advanced MRI techniques have been employed to assess iron levels in the brain, including T1‐weighted (T1‐w), T2‐weighted (T2‐w), T2* relaxometry, susceptibility‐weighted imaging (SWI), quantitative susceptibility mapping (QSM), etc.^27^ QSM and SWI are advanced iron‐deposit‐sensitive techniques that can provide quantitative and qualitative analyses of iron concentration, respectively.^28^, ^29^, ^30^ QSM calculates the magnetic susceptibility of local tissues and translates them into quantitative values that correspond to iron levels.^31^ SWI uses variations in tissue susceptibility to provide an improved contrast that reflects the iron accumulation patterns.^32^ These methods exploit the magnetic properties of iron to generate contrast in MR images, allowing for the estimation of iron concentrations in different brain regions.^33^, ^34^, ^35^, ^36^

Previous review research predominantly focused on region‐of‐interest analyses of deep gray matter structures using R2* and magnetic susceptibility mapping techniques.^37^, ^38^ However, analyses of the PFC are becoming increasingly important for studying age‐related changes in magnetic susceptibility and, consequently, iron levels. Therefore, a comprehensive systematic review of the literature on this topic is lacking. Our review aimed to summarize the current state of knowledge of assessing PFC iron content using MRI in patients with neurodegeneration and healthy subjects. We also discuss the implications of these findings for research and clinical practice.

## 2 METHODS

### 2.1 Search strategy

The search was performed in accordance with the Preferred Reporting Items for Systematic Reviews and Meta‐Analyses (PRISMA) guidelines.^39^ We conducted a thorough search method to identify relevant studies. However, this systematic review was not registered with PROSPERO or any other registry for systematic reviews. The search was performed using the PubMed, Scopus, Web of Science (WOS), and Embase databases. An updated search was conducted on studies published from inception to December 4, 2023. The search terms are listed in Supplementary Table 1. The search strategy was modified based on syntax and indexing of each database.

### 2.2 Study selection/inclusion and exclusion criteria

Three reviewers (S.G., S.M., and M.H.) independently screened the titles and abstracts of articles retrieved from the search. Full‐text articles were reviewed if they met the following inclusion criteria: (1) observational or experimental studies investigating prefrontal iron content using MR susceptibility imaging techniques; (2) studies including diseases with neurodegeneration; (3) studies including healthy human adults as participants; (4) studies reporting quantitative measures of prefrontal iron content; and (5) studies published in English with full‐text availability. Any discrepancies between reviewers were resolved through discussion and consensus. Articles that were not peer‐reviewed, not in English, were case reports, case series, or book chapters were excluded. Case reports and case series were excluded due to their inherently limited sample sizes, which reduce statistical power and generalizability, and their observational design, which increases susceptibility to bias. We only considered studies that focused on human subjects and advanced the MRI findings. We manually verified the reference lists of the selected articles. Finally, the reviewers re‐examined eligible articles to extract the data. We did not perform a meta‐analysis because of differences in the study populations, interventions, outcomes, and methodologies. Instead, we reported MRI and neuropsychological findings using narrative synthesis to help nonprofessional readers understand the results.

### 2.3 Data extraction and quality assessment

Data were extracted from the selected articles using a pre‐designed data extraction form. The following information was extracted: (1) study characteristics, (2) sample size and health status, (3) MRI techniques (such as imaging sequences), (4) PFC region(s), and (5) study outcome. To evaluate the quality and potential biases of the included studies, the Newcastle‐Ottawa Scale (NOS), derived from the Ottawa checklist for cross‐sectional studies, was utilized.^40^ This extensively acknowledged tool assesses studies based on three domains: selection, comparability, and outcome, with scores ranging from 0 to 9. A score of 9 denotes very high quality, 7–8 represents good quality, 5–6 indicates satisfactory quality, while scores between 0 and 4 reflect unsatisfactory quality. For the purpose of this review, the NOS was slightly modified in alignment with the methodology employed in two prior meta‐analyses.^41^, ^42^

## 3 RESULTS

### 3.1 Overview of results

**FIGURE 1:**
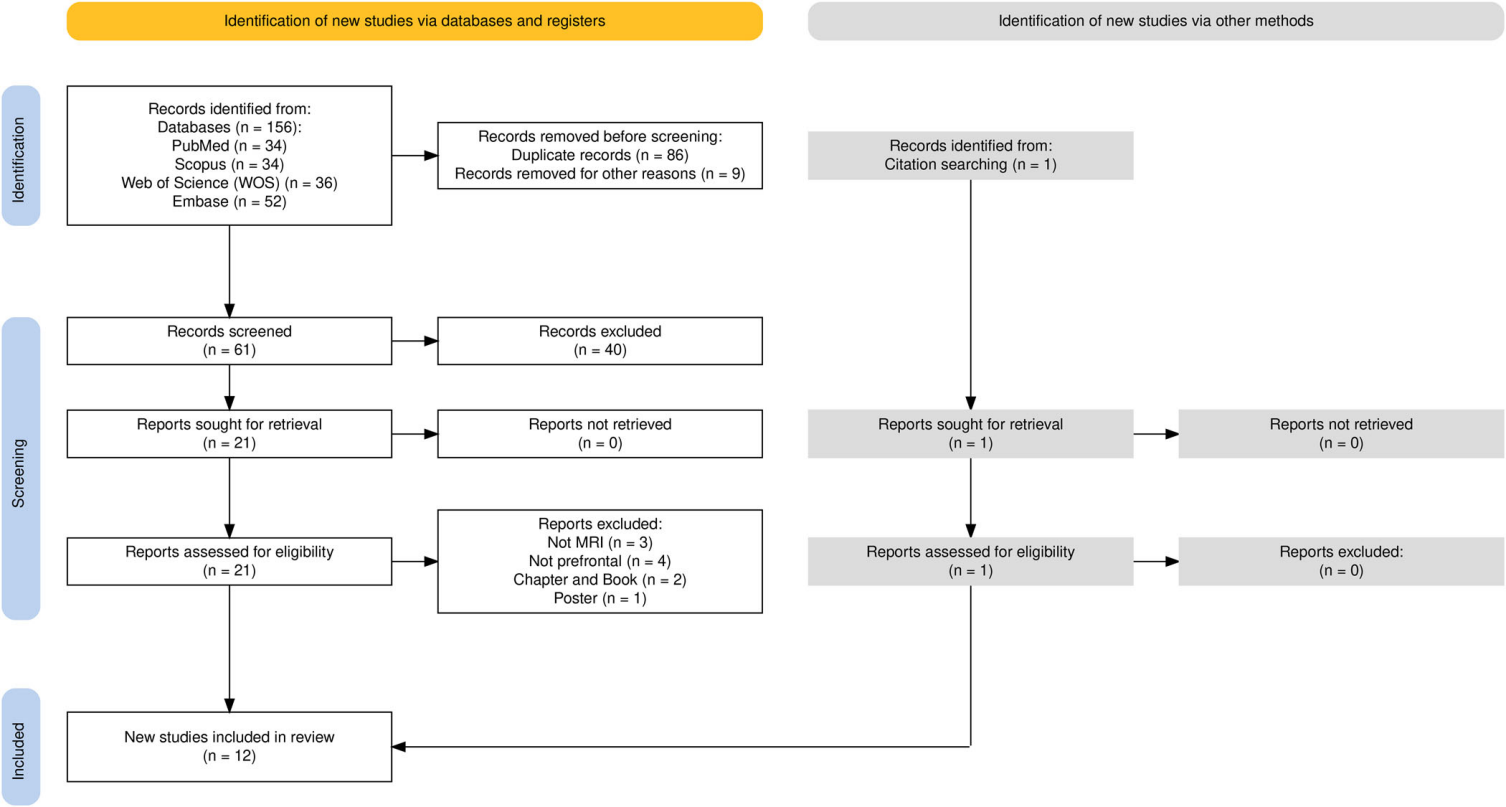
Preferred Reporting Items for Systematic Reviews and Meta‐Analyses (PRISMA) flow diagram for included studies. [Color figure can be viewed at wileyonlinelibrary.com]

Our systematic review identified 12 studies eligible for inclusion (Figure 1). The selection process followed a structured approach to ensure methodological rigor. Initially, 156 records were identified across four databases (PubMed: *n* = 34; Scopus: *n * = 34; Web of Science: *n* = 36; Embase: *n* = 52). After removing 86 duplicate records and nine excluded for other reasons (e.g., incomplete data, non‐peer‐reviewed sources), 61 records underwent title/abstract screening. Of these, 40 were excluded due to irrelevance to the research question, leaving 21 records for full‐text retrieval. During eligibility assessment, 10 of the 21 records were excluded for the following reasons: not MRI‐based (*n* = 3), not focused on prefrontal regions (*n* = 4), book chapters or conference proceedings (*n* = 2), and poster abstracts (*n* = 1). The remaining 11 records, combined with one additional record identified through citation searching (which met all eligibility criteria), resulted in 12 studies included in the final synthesis. These studies employed techniques to measure PFC iron content, particularly MRI. Five studies involved 493 patients, including three studies on PD, one on MS, and one on MCI, and seven studies involved 688 healthy individuals. According to our NOS analysis, most of these studies exhibited a low risk of bias (ROB) (Table 1). Notably, a preliminary study by Gelman et al. (1999) was considered for inclusion in our review. However, the study included fewer than ten healthy participants (six subjects), which aligns with methodological guidelines defining a case series as studies with fewer than 10 participants (e.g., the Cochrane Handbook for Systematic Reviews). This classification reflects an insufficient sample size to ensure statistical validity or generalizability of findings, leading to its exclusion from our analysis.^43^ Burger et al. (2019) conducted MRI studies but did not include advanced MR‐based techniques for measuring iron. However, their findings are closely related to those of other studies, warranting their inclusion in our report.^44^

**TABLE 1:** The Newcastle‐Ottawa Scale (NOS) adapted to the cross‐sectional studies for quality assessments results.

Author (year)	Selection				Comparability	Outcome		Total score	Quality
	Representativeness of the sample	Sample size	Non‐respondents	Ascertainment of the exposure (risk factor)	Control for important or additional factors	Assessment of the outcome	Statistical test		
Chen et al. (2023)^47^	1	0	1	1	2	1	1	7	Low ROB
Gustavsson et al. (2023)^53^	1	1	1	1	2	1	1	8	Low ROB
Pontillo et al. (2022)^49^	1	1	1	1	2	1	1	8	Low ROB
Burgetova et al. (2021)^52^	1	1	1	1	2	1	1	8	Low ROB
Griffanti et al. (2020)^45^	1	1	1	1	2	1	1	8	Low ROB
Thomas et al. (2020)^46^	1	1	1	1	2	1	1	8	Low ROB
Burger et al. (2019)^44^	1	0	1	1	2	1	1	7	Low ROB
Allen et al. (2017)^51^	1	0	1	1	2	1	1	7	Low ROB
Acosta‐Cabronero et al. (2016 (^16^	1	1	1	1	2	1	1	8	Low ROB
van Bergen et al. (2016)^48^	1	0	1	1	2	1	1	7	Low ROB
Lorio et al. (2014)^50^	1	1	1	1	2	1	1	8	Low ROB
Rodrigue et al. (2011)^17^	1	1	1	1	2	1	1	8	Low ROB

Abbreviation: ROB, risk of bias.

### 3.2 Neurodegeneration findings

Table 2 summarizes the results of iron content quantification of the PFC in diseases with neurodegeneration. Griffanti et al. (2020)^45^ and Thomas et al. (2020)^46^ elucidated the relationship between PD and alterations in microstructural integrity. In particular, these modifications were correlated with executive function test performance. These changes were accompanied by increased iron content in brain tissue compared to controls, suggesting that QSM might be an effective biomarker for early cognitive changes in PD and disease progression monitoring.^45^, ^46^

**TABLE 2:** Prefrontal cortex iron content quantification with MRI in diseases with neurodegeneration.

Study	Subjects/Controls	MRI techniques	PFC Region(s)	Findings
Chen et al. (2023)^47^	16 PD‐A, 23 PD‐nA, and 26 HCs PD	T1‐w, FLAIR, QSM, and DWI	Medical PFC	VBM and ROI‐based QSM analyses showed that PD‐A had significantly increased QSM values in the mPFC. Increased brain iron deposition in several areas of the fear circuit in PD‐A, such as medical PFC. The severity of anxiety symptoms was positively correlated with iron levels in medical PFC.
Pontillo et al. (2022)^49^	117/53 MS	T1‐w, T2‐FLAIR, QSM, and R1 relaxometry	PFC	Widespread longitudinal relaxation rate decrease associated with more limited modifications of quantitative susceptibility Reduced GM relaxation rate values were associated with increased quantitative susceptibility in the PFC.
Griffanti et al. (2020)^45^	222/68 PD	T1‐w, T2‐FLAIR, DWI, SWI, and fMRI	PFC	Alterations in the microstructural integrity of the PFC in PD patients that correlated with performance on executive function tests. Imaging of the substantia nigra with the more recent adoption of sequences sensitive to iron and neuromelanin content shows promising results in identifying early signs of Parkinsonian disease.
Thomas et al. (2020)^46^	100/37 PD	T1‐w and QSM	PFC	Increases in brain tissue iron content in the PFC of individuals with PD compared to controls. QSM can be a useful biomarker for detecting early cognitive changes in PD and monitoring disease progression.
van Bergen et al. (2016)^48^	15/22 MCI	T1‐w, QSM, and fMRI	mPFC	Significant increased iron‐associated coupling with the mPFC.

Abbreviations: DWI, diffusion‐weighted imaging; FLAIR, fluid‐attenuated inversion recovery; fMRI, functional magnetic resonance imaging; GM, gray matter; HCs, healthy controls; MCI, mild cognitive impairment; mPFC, medial prefrontal cortex; MRI, magnetic resonance imaging; MS, multiple sclerosis; PD, Parkinson's disease; PD‐A, Parkinson's disease with anxiety; PD‐nA, Parkinson's disease without anxiety; PFC, prefrontal cortex; QSM, quantitative susceptibility mapping; ROI, region of interest; SWI, susceptibility‐weighted imaging; T1‐w, T1‐weighted; T2‐FLAIR, T2‐weighted fluid‐attenuated inversion recovery; VBM, voxel‐based morphometry.

Further analysis by Chen et al. (2023)^47^ indicated that PD patients with anxiety (PD‐A) had significantly increased QSM values in the medial PFC (mPFC), as evidenced by voxel‐based morphometry (VBM) and regions of interest (ROI)‐based QSM analyses. This study also uncovered a positive correlation between anxiety symptom severity and mPFC iron levels.^47^

In MCI, Bergen et al. (2016) observed a selective increase in iron‐associated coupling in the mPFC by using combined QSM and functional MRI (fMRI).^48^ Lastly, Pontillo et al. (2022) found associations between a decreased relaxation rate and increased QSM values in the PFC, indicating potential iron accumulation.^49^

### 3.3 Healthy findings

Several studies demonstrated age‐related increases in iron content within PFC subregions, including dorsolateral PFC (DLPFC), mPFC, and anterior PFC (aPFC) in healthy participants summarized in Table 3. Regarding healthy aging, Rodrigue et al. (2011)^17^ and Lorio et al. (2014)^50^ found that age‐related differences in iron and volume within the PFC followed similar patterns,^17^ with increased volume estimation in the PFC observed in magnetization transfer (MT)‐derived gray matter volume maps. These changes were linked to the interaction between age, iron content, and probability of gray matter (GM) classification in several brain regions.^50^ Furthermore, Acosta‐Cabronero et al. (16) observed signs of iron accumulation in the superior PFC.^16^ Allen et al. (2017)^51^ found significant effects of myelination and iron in several areas, including the DLPFC, aPFC, and precuneus.

**TABLE 3:** Prefrontal cortex iron content quantification with MRI in healthy subjects.

Study	Healthy subjects	Male/female Mean ± SD [age range]	MRI	Region	Findings
Gustavsson et al. (2023)^53^	180	90/90 49.8 ± 17.4 [20–79]	T1‐w, QSM, and tb‐fMRI (N‐back task)	PFC	Elevated iron content was related to lower D1DR in DLPFC. Dopamine‐rich regions, such as the striatum, may be less susceptible to elevated iron. The combination of elevated iron load and reduced D1DR in older individuals contributes to disturbed PFC‐related circuits. Older individuals with elevated iron and lower D1DR exhibited less frontoparietal activations during demanding tasks, which was related to poorer working memory performance. The relationship between iron and the PFC suggests that elevated iron load and reduced D1DR in the PFC may contribute to disturbed PFC‐related circuits and poorer working memory performance in older individuals.
Burgetova et al. (2021)^52^	95	38/57 37 ± 10 [21–58]	T1‐w and QSM	PFC DLPFC mPFC	Linear age‐related increase in magnetic susceptibility found in DLPFC and mPFC. Highest age‐related susceptibility increases in cortical regions involved in motor (precentral, postcentral, premotor), cognitive (prefrontal, temporal, insula, precuneus), and visual (occipital, cuneus, cingulate, fusiform, lingual) functions.
Burger et al. (2019)^44^	40	20/20 M: 31.9 ± 5.2 F: 31.7 ± 7.3 [21–58]	MRS [and serum iron (µmol/L)]	DLPFC	Significant associations between peripheral iron markers and Glx:tCr found only in female group. In females, right DLPFC Glx:tCr positively associated with serum transferrin and negatively with transferrin saturation. In females, right frontal WM Glx:tCr negatively associated with iron concentration and transferrin saturation.
Allen et al. (2017)^51^	48	19/29 24 ± 4 [20–40]	R1 and R2* relaxometry	Anterior PFC	Significant effects of myelination and iron content on brain anatomy found in dorsolateral/anterior PFC and precuneus. Metacognition‐neuroanatomy relationship in dorsolateral/anterior PFC and precuneus may reflect changes in underlying myeloarchitecture rather than GM. Myelination and iron content contribute to anatomical differences in selective prefrontal and parietal regions implicated in metacognition.
Acosta‐Cabronero et al. (2016)^16^	116	60/56 54 ± 19 [20–79]	T1‐w, T2‐w, QSM, and SWI	Superior PFC	Patchy signs of iron scavenging were observed in the superior PFC.
Lorio et al. (2014)^50^	96	40/56 M: 55 ± 15 F: 57 ± 19 [M: 27–74/F: 21–88]	R1 and R2* mapping	PFC	Increased volume estimation in the PFC in MT‐derived GMV maps. Positive correlation between GMV differences (MT vs. R1) and R2* values in subcortical regions like caudate, putamen, pallidum, substantia nigra, thalamus, as well as cingulate, PFC, and dentate. Increased GM probability and volume estimated using MT vs R1 maps in basal ganglia, thalamic pulvinar, PFC and cerebellum explained by interactive effects of age and iron content.
Rodrigue et al. (2011)^17^	113	37/76 M: 58.46 ± 13.82 F: 51.76 ± 15.71 [NR]	T2* relaxometry	PFC	In PFC age‐related differences in iron and volume followed similar patterns.

Abbreviations: DLPFC, dorsolateral prefrontal cortex; D1DR, D1‐like dopamine receptors; fMRI, functional magnetic resonance imaging; F, female; GM, gray matter; GMV, gray matter volume; Glx, glutamine; MRS, magnetic resonance spectroscopy; mPFC, medial prefrontal cortex; MRI, magnetic resonance imaging; MT, magnetization transfer; M, male; NR, not record; PFC, prefrontal cortex; QSM, quantitative susceptibility mapping; SWI, susceptibility‐weighted imaging; T1‐w, T1‐weighted; T2‐w, T2‐weighted; tb‐fMRI, task‐based fMRI; tCr, total creatine.

Burger et al. (2020)^44^ reported significant associations between peripheral markers of iron metabolism and glutamine (Glx) and the concentration of total creatine (tCr) (Glx: tCr) in the right DLPFC in the female group. Furthermore, Burgetova et al. (2021)^52^ reported a linear increase in magnetic susceptibility with age in the DLPFC and mPFC. Finally, Gustavsson et al. (2023)^53^ found that elevated iron content is related to lower D1‐like dopamine receptors (D1DR) in the DLPFC. They also showed that older individuals with high iron levels and lower D1DR exhibited less frontoparietal activation during demanding tasks related to poorer working memory performance.^53^

## 4 DISCUSSIONS

This systematic review synthesized evidence from 12 studies using MRI techniques to quantify the iron content in the PFC of healthy individuals and patients with neurodegeneration, as well as the implications of these findings for cognitive and behavioral functions. These studies demonstrated the feasibility and effectiveness of using MRI to track the iron content in the PFC. The MRI techniques employed in these studies, such as SWI, QSM, T2*‐w, and R2*, allowed for accurate and reliable measurements of the iron content in the PFC, which is crucial for the potential clinical applications of this monitoring.

Our analysis of studies revealed a consistent pattern: higher iron levels in the PFC are associated with poorer cognitive performance, suggesting a crucial role for iron in age‐related cognitive decline and neurodegenerative processes.^54^, ^55^ This review demonstrates that PFC iron content could serve as a valuable biomarker for various neurological and neuropsychiatric conditions, including PD, MCI, and MS.^45^, ^46^, ^47^, ^48^, ^49^ Increased iron content in the PFC might be an early marker of cognitive impairment in patients with PD^45^ and a valuable biomarker for detecting early cognitive changes and monitor disease progression.^46^ The association between anxiety in PD and increased iron levels in the fear circuitry^47^ suggests that iron content in the PFC may play a role in the neural mechanisms underlying anxiety in PD. Furthermore, increased iron‐associated coupling with the mPFC was observed in subjects with MCI,^48^ and longitudinal relaxation rate decreases were found in patients with MS.^49^ The results of studies on MCI^48^ and MS^49^ further support the potential role of iron content in the PFC in the pathophysiology of these conditions. This finding aligns with the known vulnerability of the PFC to iron deposition and its critical role in cognitive functions, such as attention, working memory, and decision‐making.^21^, ^56^

Studies on diseases with neurodegeneration have consistently found a higher iron content in the PFC subregions of the patient groups than in controls, as measured by QSM. The areas affected include the mPFC in patients with anxiety,^47^ the mPFC in MCI,^48^ and the PFC in MS.^49^ This finding may indicate neuroinflammation, oxidative stress, or neuronal loss under these conditions.^46^, ^47^, ^57^ Executive functions, such as working memory and attention, often associated with PFC dysfunction, can be altered according to the pattern of increased iron deposition.^19^, ^45^, ^58^ This suggests that iron dyshomeostasis is likely a contributing factor to the pathophysiology underlying the cognitive‐behavioral symptoms in these disorders.^46^, ^47^, ^59^

Various factors influence the PFC in healthy individuals, including age,^17^, ^50^, ^52^ sex,^44^ peripheral iron metabolism,^44^ and dopamine receptor density.^53^ Studies have consistently shown that iron content gradually increases in healthy adults, possibly due to normal aging or subclinical neurodegeneration.^5^, ^17^, ^60^ Another study also found a link between elevated iron content and lower D1DR in the DLPFC, suggesting a connection between iron dysregulation and dopaminergic dysfunction in the aging brain.^53^, ^61^ Dopamine plays a crucial role in cognitive function, and its decline with age is a well‐established phenomenon. The observed association between iron and dopamine suggests that iron accumulation could exacerbate age‐related cognitive decline by further impairing dopaminergic neurotransmission.^62^, ^63^

Studies on healthy aging have consistently observed an increase in iron content with age, affecting specific subregions such as the DLPFC, mPFC, and aPFC.^44^, ^51^, ^52^ Higher iron levels are associated with reduced dopamine receptor expression and function and poorer working memory performance, indicating that iron accumulation contributes to age‐related cognitive decline.^53^, ^64^ Higher PFC iron levels are associated with normal aging and may be a key process affecting structure and cognition.^51^, ^65^ The relationship between iron levels and brain structure, myeloarchitecture, and cognitive function is complex, particularly in regions implicated in metacognition and working memory.^17^, ^50^, ^51^, ^53^ Understanding this relationship is crucial to address age‐related cognitive decline.

Two studies highlighted QSM as a potential diagnostic and monitoring biomarker in PD^46^, ^47^ based on its sensitivity to early microstructural alterations linked to cognitive decline. Regular tracking of PFC iron levels using QSM can inform disease screening, diagnosis, and progression. This indicates that QSM could be a reliable biomarker for monitoring iron deposition‐related cognitive changes in the early stages of disease progression.^66^, ^67^ Notably, QSM studies have shown that age‐related iron accumulation extends to the frontal lobes, suggesting a possible link between iron deposition and age‐related decline in motor and output functions.^16^, ^27^

This review supports the hypothesis that iron accumulation in the PFC is a common feature in neurodegenerative and cognitive aging diseases. Although iron is essential for normal brain function, excessive iron levels can cause oxidative damage and neurodegeneration. The PFC is particularly vulnerable to iron overload owing to its high metabolic demand, late myelination, and rich dopaminergic innervation. Accumulation in the PFC may affect its integrity and function, which are responsible for higher‐order cognitive processes such as working memory, planning, inhibition, and emotion regulation. However, the precise mechanisms underlying the association between PFC iron accumulation and cognitive decline remain unclear. However, several hypotheses have been proposed, including iron‐induced oxidative stress, mitochondrial dysfunction, and disruption of neuronal signaling pathways.^68^

While elevated iron levels are associated with poorer cognitive function, it is important to note that QSM signals are influenced by both paramagnetic (e.g., iron) and diamagnetic (e.g., myelin) sources. Therefore, future research should investigate the relative contributions of iron deposition and myelin reduction using subvoxel techniques^69^, ^70^ to age‐related changes in PFC QSM signal intensity and cognitive decline, especially considering the potential plateau in iron levels after age 30.^1^ Furthermore, although iron accumulation is often viewed as a hallmark of aging and neurodegeneration, it is important to consider the complex interplay of iron with other factors such as brain volume changes.^17^, ^50^ For instance, in medial temporal structures, such as the hippocampus and entorhinal cortex, age‐related volume reduction does not always align with changes in iron content.^17^, ^71^ This highlights the need for further investigation of the intricate relationships between iron, brain structure, and cognitive function.

It is important to consider certain limitations when analyzing the results of this review. Larger and more diverse groups of diseases with neurodegeneration should be studied to draw generalized conclusions. Moreover, most studies were not conducted over long periods, which limits their ability to determine trends. Several implications for research and clinical practice should be considered. There are standardized protocols and methods for measuring iron content using MRI techniques, such as the novel QSM pipeline.^72^ Different MRI techniques may have varying degrees of sensitivity and specificity for detecting the iron content in other parts of the brain. In addition, different image acquisition parameters, processing pipelines, and analysis methods can introduce bias and variability in the estimation of the iron content.

## 5 CONCLUSIONS

In conclusion, our findings consistently highlight the significance of the iron content in the PFC in both healthy aging and neurodegeneration. Advanced MRI techniques, particularly QSM, are valuable for mapping and quantifying these alterations. These studies have consistently demonstrated a correlation between elevated iron levels in the PFC and diminished cognitive function. Our findings suggest that elevated PFC iron levels may serve as valuable imaging biomarkers for neurodegeneration and cognitive decline. Longitudinal studies integrating advanced QSM and other neuroimaging techniques with cognitive assessments are essential to further elucidate the impact of iron dysregulation on PFC function. This study trajectory holds promise for enhancing our understanding of the pathogenesis of neurodegeneration and the effects of aging on the brain.

## AUTHOR CONTRIBUTIONS

Sadegh Ghaderi and Sana Mohammadi contributed to writing the first draft, refining the ideas, analyzing most of the data, and creating figures and tables. Sadegh Ghaderi and Farzad Fatehi put forward a central idea and finalized this study. Sadegh Ghaderi, Sana Mohammadi, and Masoud Hoseini Pourasl contributed to the literature searching and literature screening.

## CONFLICT OF INTEREST STATEMENT

The authors declare no conflicts of interest.

## ETHICS STATEMENT

Not applicable.

## Supporting information

Supplementary File.

## Data Availability

This article contains all the data produced or analyzed during this investigation. Further inquiries should be forwarded to the corresponding author.
