# Supplementary material for: Prefrontal cortex iron content in neurodegeneration and healthy subjects: A systematic review
Source: Ibrain. 2025 Apr 10;11(2):215–27. doi: 10.1002/ibra.12195 (PMC12177664; doi:10.1002/ibra.12195)
Supplement: Supplementary file 1 — Supplementary File. [file IBRA-11-215-s001.docx]

| **Supplementary Table 1.** The search strategies used for database searches | | |
| --- | --- | --- |
|  | **Database** | **N** |
| PubMed | ("prefrontal*"[Title/Abstract]) AND ("iron"[Title/Abstract] OR "susceptibili*"[Title/Abstract] OR "χ"[Title/Abstract]) AND ("SWI"[Title/Abstract] OR "susceptibility weighted imag*"[Title/Abstract] OR "susceptibility-weighted imag*" OR"quantitative susceptibility*"[Title/Abstract] OR "T2-w"[Title/Abstract] OR "R2"[Title/Abstract] OR "relaxom*"[Title/Abstract]) | 34 |
| Scopus | TITLE-ABS("prefrontal*" AND ("iron" OR "susceptibili*" OR "χ") AND ("SWI" OR "susceptibility weighted imag*" OR "susceptibility-weighted imag*" OR "quantitative susceptibility*" OR "T2-w" OR "R2" OR "relaxom*")) | 34 |
| Web of Science (WOS) | TS=(("prefrontal*" AND ("iron" OR "susceptibili*" OR "χ") AND ("SWI" OR "susceptibility weighted imag*" OR "susceptibility-weighted imag*" OR "quantitative susceptibility*" OR "T2-w" OR "R2" OR "relaxom*"))) | 36 |
| Embase | ('prefrontal*' AND ('iron' OR 'susceptibili*' OR 'χ') AND ('SWI' OR 'susceptibility weighted imag*' OR 'susceptibility-weighted imag*' OR 'quantitative susceptibility*' OR 'T2-w' OR 'R2' OR 'relaxom*')):ti,ab | 52 |
